# Diagnostic role of 18F-FDG PET/MRI in patients with gynecological malignancies of the pelvis: A systematic review and meta-analysis

**DOI:** 10.1371/journal.pone.0175401

**Published:** 2017-05-08

**Authors:** Ji Nie, Jing Zhang, Jinsheng Gao, Linghong Guo, Hui Zhou, Yuanyuan Hu, Chenjing Zhu, Qingfang Li, Xuelei Ma

**Affiliations:** 1 State Key Laboratory of Biotherapy and Cancer Center, West China Hospital, Sichuan University, Chengdu, PR China; 2 Department of Neurosurgery, West China Hospital, Sichuan University, Chengdu, PR China; 3 Oncology Department, Yilong County People’s Hospital, Chengdu,PR China; National Cancer Centre Singapore, SINGAPORE

## Abstract

**Purpose:**

The aim of this study was to assess the diagnostic performance of 18F-FDG Positron Emission Tomography/Magnetic Resonance Imaging (PET/MRI) for gynecological cancers of the pelvis, based on a systematic review and meta-analysis of published data.

**Methods:**

We performed a comprehensive literature search of Pubmed and Embase for studies that evaluated the diagnosis of 18F-FDG PET/MRI for gynecological malignancies in the pelvis. Quality Assessment for Studies of Diagnostic Accuracy 2 (QUADAS 2) tool was used to access the quality of included studies. After testing heterogeneity of the pooled studies with I^2 and H^2 (calculated using metaan in Stata12.0) we treated the data that extracted and transformation from the studies, based on DerSimonian-Laird method(Random-effects models),then back-transformation them to percentages and plotting to get the pooled sensitivity, specificity, likelihood ratios, and constructed summary receiver operating characteristics (SROC) curve.

**Results:**

Eventually, 7 studies fulfilled our predefined inclusion criteria were included in our research. On patient-based assessment, the pooled sensitivity, specificity, positive likelihood ratio, negative likelihood ratio and diagnostic odds ratio of 18F-FDG PET/MRI for diagnosis of gynecological malignancies were 0.95 (95%CI 0.86–0.99), 0.95 (95% CI 0.74–1.00), 7.51 (95% CI 2.29–24.59), 0.12 (95% CI 0.05–0.29) and 116.27 (95% CI 17.07–791.74), respectively. On lesion-based assessment, the pooled sensitivity, specificity, positive likelihood ratio, negative likelihood ratio and the summary DOR were 0.89 (95%CI 0.84–0.93), 0.87 (95%CI 0.74–0.95), 6.99 (95%CI 3.30–14.79), 0.12 (95%CI 0.06–0.25) and 55.82 (95%CI 20.91–149.05), respectively.

**Conclusions:**

Our meta-analysis indicated that 18F-FDG PET/MRI, combined the advantages of MRI and PET, may be a very promising diagnostic method to assess the primary tumor and nodal staging in patients with gynecological malignancies of the pelvis.

## Introduction

Gynecological malignancies in the pelvis, mainly including cervical carcinoma, ovarian cancer, and endometrial cancer, is a sever threat to women’s health and life. Over a million people are diagnosed with gynecological cancers and half million people are dead per year [1, 2]. Up to now, early surgical intervention is still the principle of management for patients with gynecological pelvic malignancies, and appropriate surgical planning is highly depended on staging and restaging of tumors. Therefore, high quality imaging assessment of gynecological malignancies is essential to the best feasible patient management and therapy [3–5]. For the past few years, imaging methods for gynecological cancers mainly included ultrasound, X-ray, CT and MRI, while none of them achieved satisfying diagnostic value. Inline positron emission tomography (PET)/computed tomography (CT) is now reported by quite a few studies as a powerful imaging modality for different gynecological cancers [6–10]. However, due to its low soft-tissue contrast, CT imaging reveals limitation in precise assessment of potential tumor infiltrating into surrounding tissue, especially in the pelvis. Also, it triggers an increased radiation dose which can lead to a potential harm to the patient. Thus, more accurate imaging examination methods need to be found.

Fused PET/MRI (positron emission tomography/ magnetic resonance imaging), the combination of MR imaging and PET in a single machine, is now suggested for detection of malignancies in many sites, especially in soft tissues such as pelvis. Similar to PET/CT, PET/MRI is capable to provide metabolic data based on the PET component. PET/MRI also provides excellent soft tissue contrast under the avoidance of ionizing radiation exposure [11–16]. Thus, fused PET/MRI has been introduced and developed recently. But its clinical application and diagnostic value in the gynecological disease still needs to be clarified. Until now, integrated PET/MRI has been reported to be high diagnostic in the evaluation of gynecological tumor entities in some studies. However existing studies are inconclusive because of a relatively small sample size. Also, the quality of these studies has not been assessed systematically [17–19].

We performed this meta-analysis to systemically review all relevant publications and evaluate the overall accuracy and diagnostic value of PET/MRI in patients with gynecological malignancies of the pelvis.

## Methods

### Search strategy

A comprehensive literature search was performed to find relevant published articles about the diagnostic value of PET/MRI in patients with gynecological malignancies of the pelvis. We used combinations of following keywords: (a) ‘PET/MRI’ or ‘PET/MR’ and (b) ‘PET-MRI’ or ‘PET-MR’ and (c) ‘carcinoma’ or ‘cancer’. PubMed and EMBASE, from January 1990 to February 2016, were searched with no language restrictions. To maximize the search result, references of the retrieved articles were also screened for additional studies.

### Study selection

After removing duplicates of the retrieved articles, two reviewers read all the abstracts for eligibility independently. Disagreements were resolved by consensus. The same two researchers independently assessed the full-text of potentially eligible studies. Disagreements were resolved by consensus. The inclusion criteria were: (1) Studies investigated the performance of PET/MRI in patients with gynecological malignancies of the pelvis; (2) Studies used histopathology analysis and/or clinical and imaging follow-up as the reference standard; (3) Articles involved sufficient data to construct or calculate the absolute numbers of true-positives (TP), false-positives (FP), true-negatives (TN), false-negatives (FN); (4) Articles with the most sufficient details or the latest articles when data were presented in more than one article; (5) Review articles, editorials, letters, comments, conference proceedings, case reports, preclinical studies and animal studies were excluded.

### Data extraction

Two independent investigators extracted the data needed from the selected studies, with disagreements resolved by consensus. For each article, information of the principal author, year of publication, patient characteristics, inclusion criteria, reference standard used, as well as criteria used to define the cut-off between positive and negative PET/MRI was collected. The number of true positives (TP), false positives (FP), true negatives (TN), and false negatives (FN) was extracted directly or recalculated if necessary. For some studies that didn’t provide enough data, we tried to contact the authors.

### Quality assessment

We estimated the quality of the eligible studies in this meta-analysis by using the quality assessment tool for diagnostic accuracy studies (QUADAS-2) [20]. This system is composed of 4 parts that evaluate the quality of studies, especially investigations of diagnostic accuracy [21]. Each item may be assessed as 'yes (high quality)', 'no (low quality)' or 'unclear(no adequate information provided)'. The quality assessment was done by two researchers independently, and disagreements were resolved by consensus. QUADAS 2 was performed with Review Manager 5.2.

### Statistical methods

Based on the data extracted from each individual studies, we constructed 2×2 contingency tables value for TP, FP, TN, and FN to calculate the pooled sensitivity, specificity, positive (LR+) and negative (LR-) likelihood ratios and diagnostic odds ratio (DOR) (with corresponding 95%CI), using DerSimonian-Laird method(Random-effects models) in Meta-DiSc statistical software, version 1.4 (Unit of Clinical Biostatistics, Ramony Cajal Hospital, Madrid, Spain) [22–23]. The inconsistency index (*I*2) and H^2 were assessed to test heterogeneity of the pooled studies (calculated using metaan in Stata12.0) [24–25]. The summary receiver operating characteristic curve (SROC) was constructed by using the derived estimates of sensitivity, specificity, and respective variances. If there was threshold effect on the SROC curve, the maximum joint sensitivity and specificity (Q index) and the area under the SROC (AUC) could be used to assess the overall accuracy of diagnostic test [26].

## Results

### Study selection and characteristics

**Fig 1:**
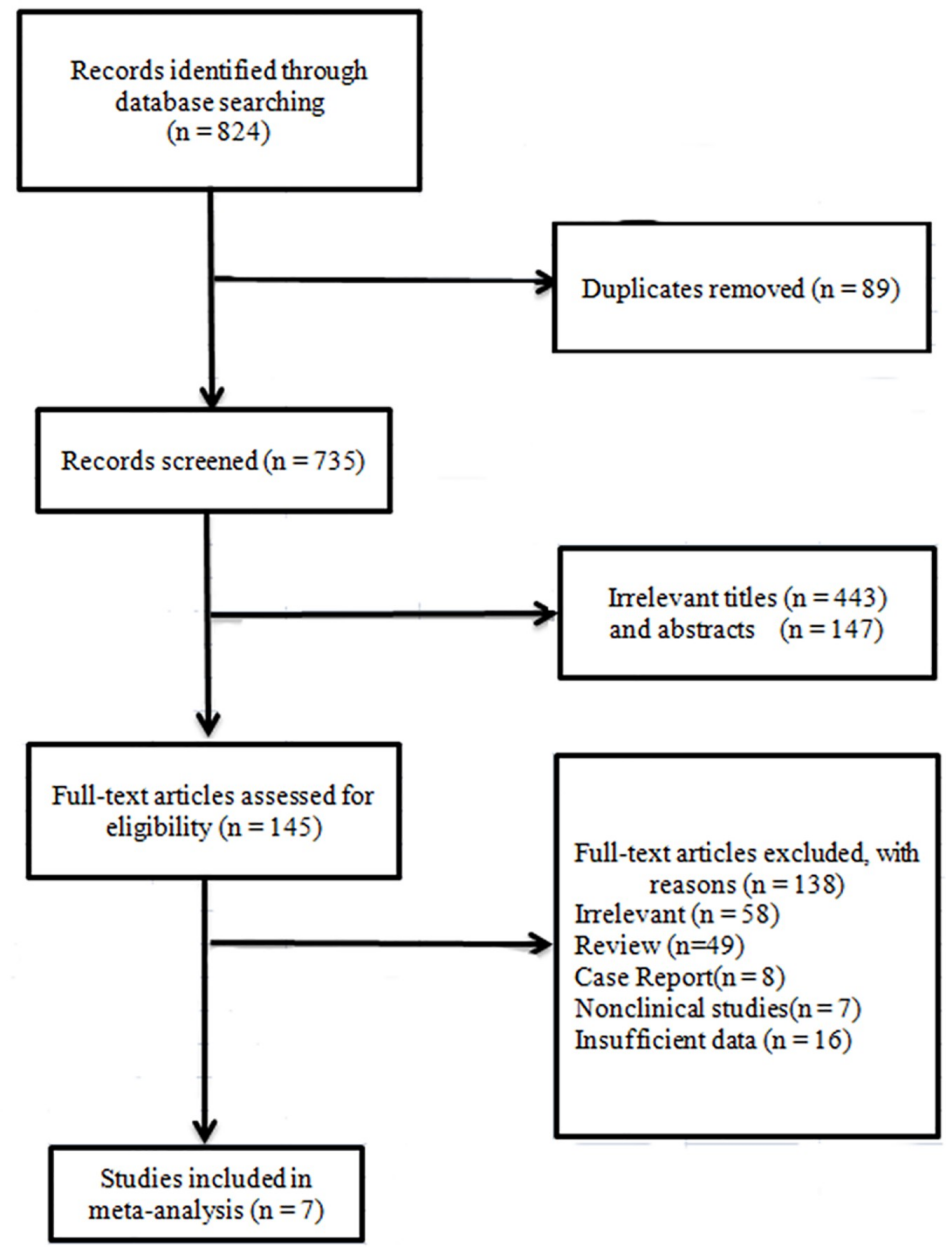
Flow chart of literature search and selection schema.

The selection and inclusion process for this meta-analysis is presented in Fig 1. After removing duplicates from the initially identified 824 relevant studies, 735 studies were screened. Then we excluded another 590 ineligible articles through the titles and abstracts. The remaining 145 candidate studies were downloaded and assessed for eligibility, and 138 articles were further removed. Eventually, seven studies were eligible in our research [27–33]. There were two authors each one of whom has published three articles, the patient samples of these studies with the same author(s) were not completely same and they presented different data concerning subgroup analysis. Consequently, all these six studies were included in our research.

The general characteristics of the studies involved in this research are shown in Table 1. Three studies reported the patient-based assessment, two studies focused on the lesion-based assessment. The incidence of metastasis or invasion was analyzed in three studies.

**Table 1:** The pooled results of metastasis and invasion of 18F-FDG PET/MRI for gynecological malignancies in different sites.

Study	Year	Country	Number of Patients	Mean Age	Type of Tumor	Study Design	Consecutive Enrollment	Imaging	Reference Standard
Grueneisen J 2014[27]	2014	Germany	48	52.8(26–73)	pelvic malignancy	Prospective	Yes	PET/MRI	Histology and follow-up
Grueneisen J 2015[28]	2015	Germany	27	48(28–73)	cervical cancer	Prospective	Yes	PET/MRI	Histology and follow-up
Grueneisen J 2015[29]	2015	Germany	24	57(27–74)	pelvic malignancy	Retrospective	ND	PET/CT,PET/MRI	Histology and follow-up
Kitajima K 2014[30]	2014	Japan	30	61.3(38–83)	uterine cervical cancer	Retrospective	Yes	PET/CT,PET/MRI	Histology and follow-up
Kitajima K 2013[31]	2013	Japan	30	62.4(30–88)	endometrial cancer	Retrospective	ND	PET/CT,PET/MRI	Histology and follow-up
Queiroz MA 2015[32]	2015	Switzerland	26	60(37–81)	gynecological malignancy	Prospective	Yes	PET/CT,PET/MRI	Histology and follow-up
Kitajima K2014[33]	2014	Japan	30	57.8(27–88)	gynecological malignancy	Retrospective	Yes	PET/CT,PET/MRI	Histology and follow-up

ND: not documented.

### Quality assessment

In this meta-analysis, the quality of the included studies was assessed with QUADAS-2, the detailed information and scores were presented in S1 Fig.

### Patient-based assessment

**Fig 2:**
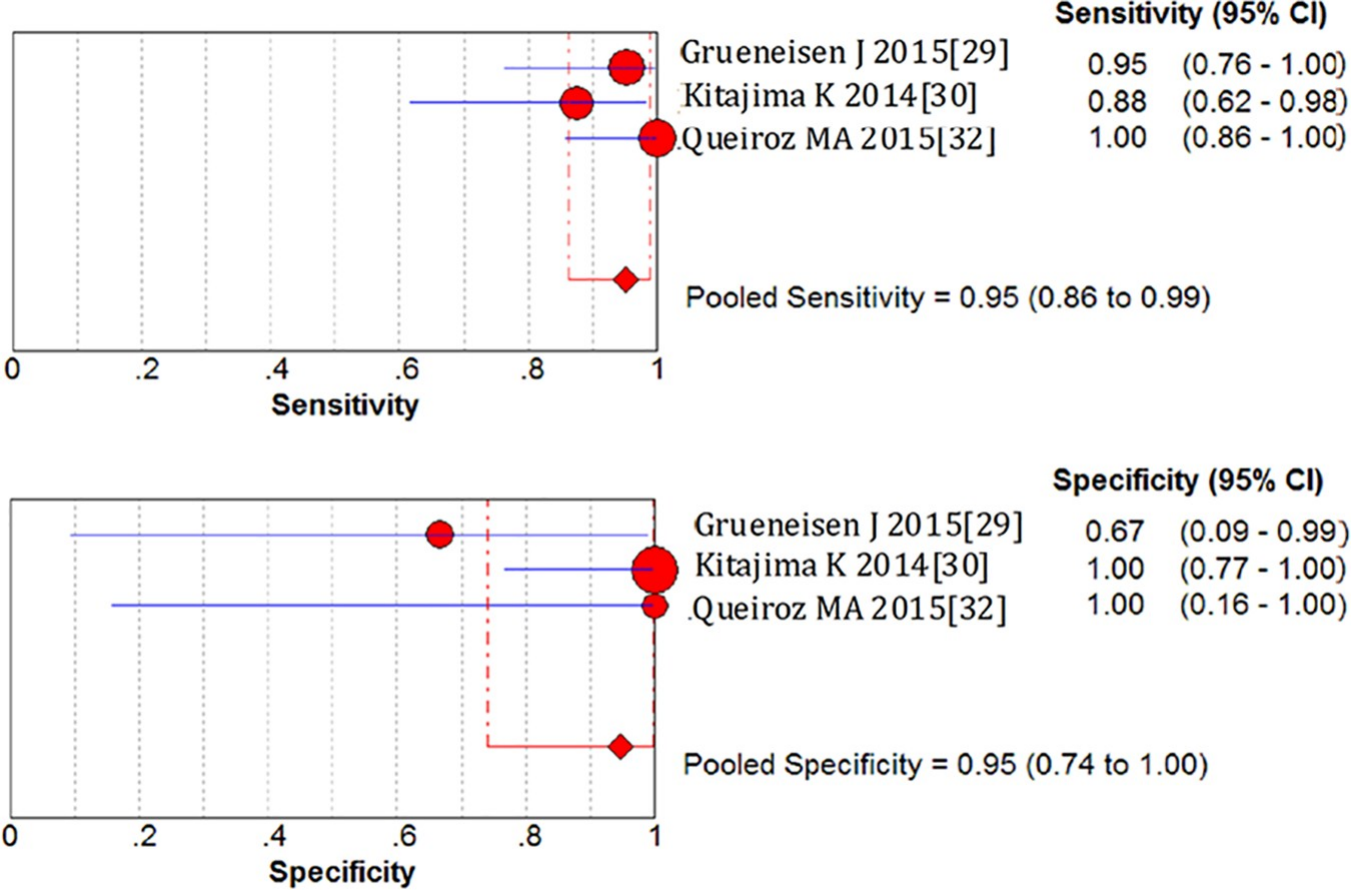
Forest plots of sensitivity and specificity for the 18F-FDG PET/MRI in the patient-based assessment of gynecological tumors.

**Fig 3:**
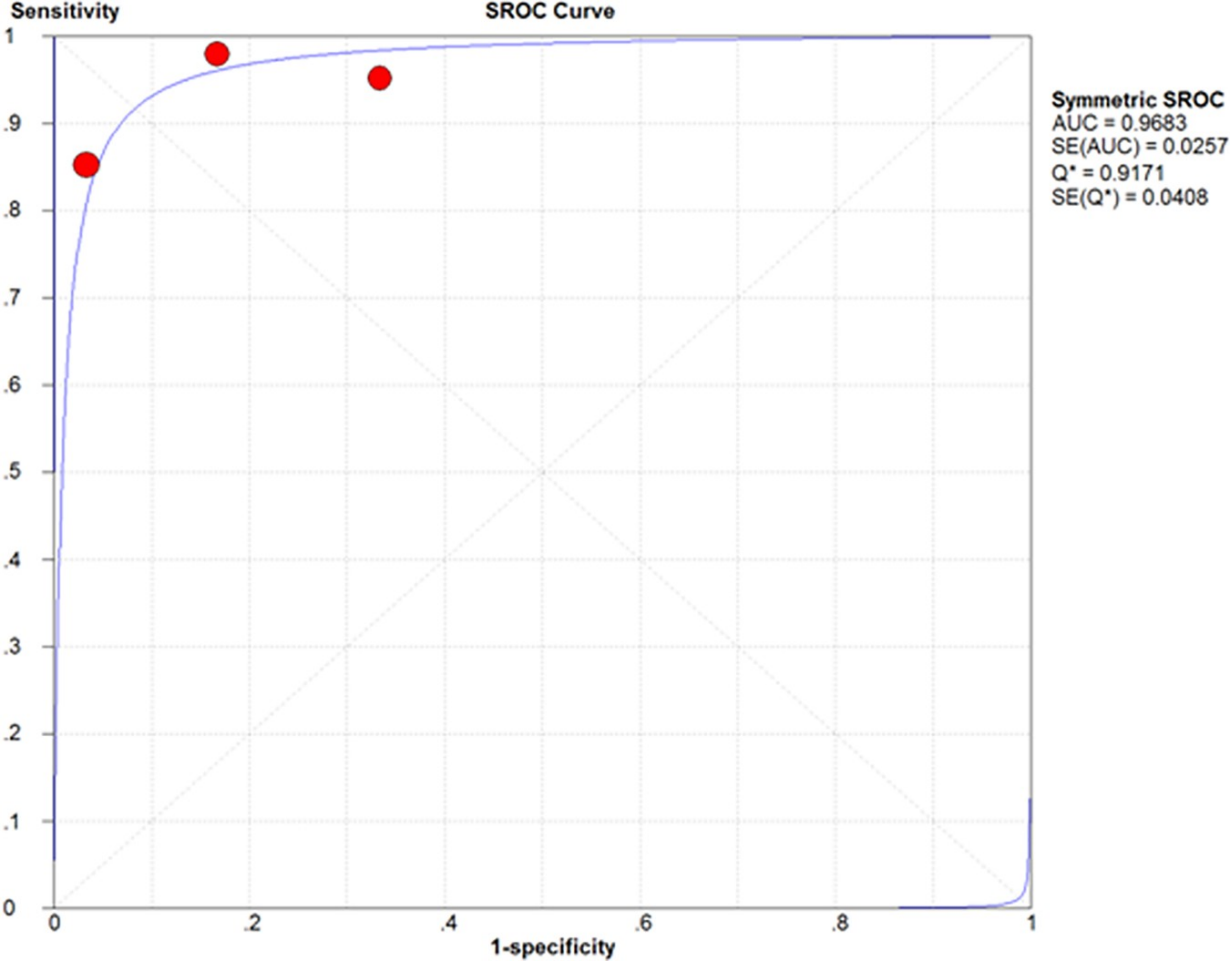
Summary receiver operating characteristic (SROC) curve for the 18F-FDG PET/MRI in the patient-based assessment of gynecological tumors.

On a per-patient basis analysis, the pooled sensitivity and specificity of 18F-FDG PET/MRI in gynecological malignancies were 0.95 (95%CI 0.86–0.99) and 0.95 (95%CI 0.74–1.00), which manifested a high diagnostic value of 18F-FDG PET/MRI. LR+ was 7.51 (95%CI 2.29–24.59) and LR- was 0.12 (95%CI 0.05–0.29). The diagnostic odds ratio (DOR) was 116.27 (95%CI 17.07–791.74) (Fig 2). Based on the sensitivity and specificity, we established an SROC curve, which indicates sensitivity versus 1-specificity of individual studies, the intersection point of the SROC curve, Q-value, corresponds to the highest common value of sensitivity and specificity for the test, showed the level of overall accuracy. As Our data showed that the overall area under the curve (AUC) of SROC was 0.9683 (standard error 0.0257), indicating that the level of overall accuracy was high (Fig 3).

### Assessment of metastasis or invasion

The TNM stage of patients has the greatest impact on prognosis and treatment planning, and the status of metastasis and invasion are the main factors that significantly determine the TNM stage. Thus, we performed the overall assessments of metastasis and invasion of 18F-FDG PET/MRI for gynecological malignancies in different sites. The results were presented in Table 2, including sensitivity, specificity, LR+, LR- and DOR. The sensitivity of 18F-FDG PET/MRI in assessment of lymph node metastasis, pelvic lymph node metastasis, parametria invasion, vagina invasion, pelvic sidewall invasion and bladder/rectum were 0.85 (95%CI 0.68–0.95), 0.94 (95%CI 0.70–1.00), 0.92 (95%CI 0.73–0.99), 0.86 (95%CI 0.57–0.98), 1.00 (95%CI 0.59–1.00) and 1.00 (95%CI 0.29–1.00), respectively. Besides, the specificity were 0.96 (95%CI 0.89–0.99), 0.93 (95%CI 0.81–0.99), 0.94 (95%CI 0.85–0.98), 0.98 (95%CI 0.88–1.00), 1.00 (95%CI 0.93–1.00) and 1.00 (95%CI 0.93–1.00), respectively. The present meta-analysis has shown that the maximum joint sensitivity and specificity (Q value) and the AUC was high for most locations of metastasis and invasion (the AUC of lymph node metastasis was as high as 0.9688 and the Q value was 0.9180), indicating a very good overall accuracy in the diagnosis of metastasis and invasion, although not perfect.

**Table 2:** The general characteristics of the studies involved in this research

		sensitivity (95% CI)	specificity (95% CI)	PLR (95% CI)	NLR (95% CI)	DOR (95% CI)	AUC	SE(AUC)	I^2 (95% CI)	H^2
patient-based assessment		0.95 (0.86–0.99)	0.95 (0.74–1.00)	7.51 (2.29–24.59)	0.12 (0.05–0.29)	116.27 (17.07–791.74)	0.9683	0.0257	1.00(1.00–1.00)	3347.95
lesion-based assessment		0.89 (0.84–0.93)	0.87 (0.74–0.95)	6.99 (3.30–14.79)	0.12 (0.06–0.25)	55.82 (20.91–149.05)	/	/	1.00(1.00–1.00)	2729.21
Metastasis	
	lymph node	0.85 (0.68–0.95)	0.96 (0.89–0.99)	16.39 (5.86–45.84)	0.18 (0.08–0.37)	90.49 (21.03–389.33)	0.9688	0.0390	1.00(1.00–1.00)	2919.25
	pelvic lymph node	0.94 (0.70–1.00)	0.93 (0.81–0.99)	9.36 (3.25–26.91)	0.10 (0.02–0.47)	98.76 (12.92–754.69)	0.5000	0.0000	1.00(1.00–1.00)	16800.44
	abdominal	1.00	1.00	/	/	/	/	/	/	/
	bone	1.00	1.00	/	/	/	/	/	/	/
Invasion	
	Parametria	0.92 (0.73–0.99)	0.94 (0.85–0.98)	8.15 (3.44–19.32)	0.12 (0.04–0.38)	85.03 (15.03–481.17)	0.9591	0.0263	1.00(1.00–1.00)	1707
	Vagina	0.86 (0.57–0.98)	0.98 (0.88–1.00)	17.87 (3.80–84.08)	0.17 (0.05–0.55)	97.79 (11.52–829.84)	0.5000	0.0000	1.00(1.00–1.00)	243.27
	pelvic sidewall	1.00 (0.59–1.00)	1.00 (0.93–1.00)	46.33 (6.51–329.47)	0.11 (0.02–0.72)	406.70 (23.70–6979.77)	0.5000	0.0000	1.00(1.00–1.00)	2090.19
	bladder/rectum	1.00 (0.29–1.00)	1.00 (0.93–1.00)	43.96 (5.70–339.15)	0.20 (0.04–1.17)	217.92 (11.80–4022.81)	0.5000	0.0000	1.00(1.00–1.00)	664.66
	Myometrial	0.90	0.78	/	/	/	/	/	/	/
	cervical stroma	0.57	1.00	/	/	/	/	/	/	/
	Adnexa	/	1.00	/	/	/	/	/	/	/
	Peritoneal	0.80	1.00	/	/	/	/	/	/	/
	uterine serosa	0.50	1.00	/	/	/	/	/	/	/

### Lesion-based assessment

**Fig 4:**
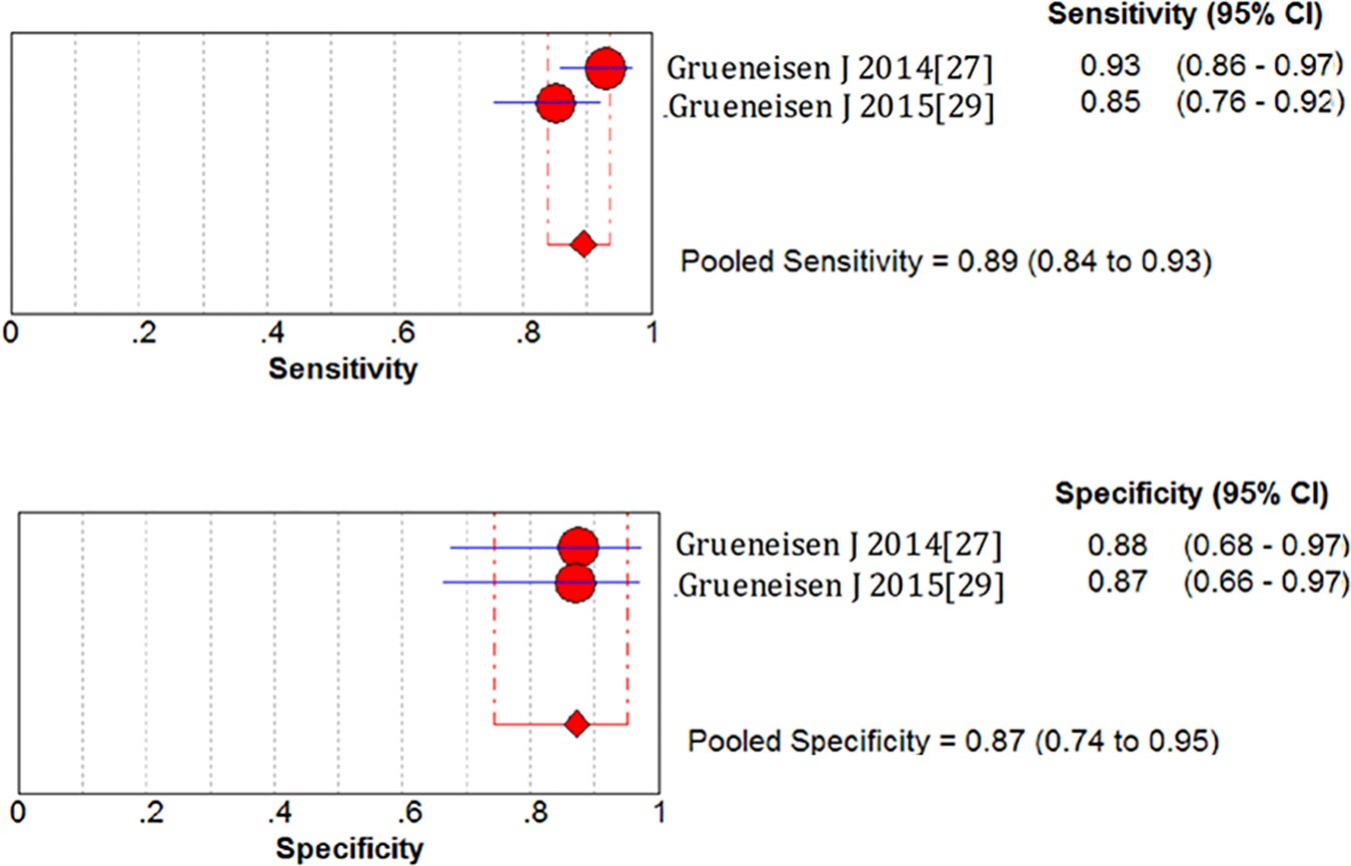
Forest plots of sensitivity and specificity for the 18F-FDG PET/MRI in the lesion-based assessment of gynecological tumors.

To achieve best management of patients, all the lesions of a patient need to be detected. Therefore, we performed the pool analysis of lesion-based assessment of 18F-FDG PET/MRI. Meta-analysis revealed that the overall sensitivity, specificity, positive likelihood ratio, negative likelihood ratio and of lesion-based assessment were 0.89 (95%CI 0.84–0.93), 0.87 (95%CI 0.74–0.95), 6.99 (95%CI 3.30–14.79) and 0.12 (95%CI 0.06–0.25) (*Fig 4*). The summary DOR was 55.82 (95%CI 20.91–149.05). Distribution of lesions in the studies was shown in S1 Table.

## Discussion

Up to now, PET/CT has been used widely in clinical diagnosis of gynecological cancers. Meanwhile, some early researches of PET/MRI showed a better performance for gynecological cancers than PET/CT. However, due to a relatively small number of patients, previous studies were limited to draw persuasive conclusions. Thus, we performed this meta-analysis and got the pooled results. To the best of our knowledge, this study is the first meta-analysis to evaluate the role of 18F-FDG PET/MRI in gynecological malignancies.

On patient-based analysis, the pooled results of our meta-analysis suggested that 18F-FDG PET/MRI had a high diagnostic value in the evaluation of patients with gynecological cancers. Three studies with a total of 60 patients were included in this meta-analysis. The pooled results of sensitivity, specificity, LR+, LR- and the AUC implied that PET/MRI had an outstanding performance for assessment of metastasis to these sites [26, 34]. These results were in line with the results of studies included in this meta-analysis and previously published data [27, 30]. To achieve the best patient management for gynecological cancers, high-quality imaging technique of early detection and evaluation is essential. 18F-FDG PET was demonstrated to be overwhelmingly accurate in pelvic malignancies detection, based on enhanced metabolic activity compared with surrounding tissue [35–37]. Functional information, such as biological aggressiveness and histological grade, can be revealed by 18F-FDG uptake after intravenously injection [38]. Compared with CT, MRI provides anatomic information of excellent soft-tissue contrast with markedly reduced radiation exposure, which is especially crucial for the management of pelvic carcinoma patients who receive radiotherapy. PET/MRI was reported to be with a significantly higher overall accuracy (83.3%) for T-staging of primary cervical cancers than PET/CT (53.3%), due to the excellent soft-tissue-contrast of the MRI component.

Actually, MRI has been demonstrated to be superior for the initial staging and assessment of recurrent gynecological cancers compared with conventional imaging methods [39–41]. Besides, PET/MRI can overcome the hinder caused by post therapeutic fibrosis and tissue scarring in pelvic soft tissue after therapy, which significantly improves the detection of recurrent diseases. Therefore, integrated 18F-FDG PET/MRI, combines the metabolic analysis based on PET with high-resolution anatomical information based on MRI, performs a high diagnostic value in patients with gynecological cancers. This is accordance with the results of our study.

According to our results, PET/MRI revealed a high diagnostic confidence in the evaluation of tumor metastasis, especially for tumor invasion into adjacent anatomical structure. In various region including pelvic sidewall and bladder/rectum, the pooled sensitivity and the pooled specificity of 18F-FDG PET/MRI achieved 1.00, implied that PET/MRI had an outstanding performance for assessment of metastasis to these sites [34]. Our results are consistent with previous published data. MRI was reported to be highly effective for assessment of gynecological cancers, especially for tumor extension and invasion [42]. Moreover, MRI has been demonstrated superior in the assessment of tumor invasions in adjacent structures [43, 44]. One study [30] included in our research compared the performance of pelvic PET/CT, MRI and 18F-FDG PET/MRI for assessment of nodal metastasis and locoregional extension of cervical cancer. The result suggested that fused PET/MRI had the equivalent T staging ability as MRI, and the same high N staging ability as PET/CT. Combined the advantages of PET and MRI, PET/MRI achieved an excellent diagnostic performance of lymph node metastasis. Thus, it provided a specific GTV (gross tumor volume) which is critical to chemotherapy of the cervical cancer patients.

The results based on lesion analysis demonstrated a relatively high diagnostic competence of 18F-FDG PET/MRI. A total of 72 patients with 226 lesions in two studies were included. The overall sensitivity, specificity and the DOR were relatively high. They were not as satisfactory as the results of patient-based assessment. Two recently published studies demonstrated that 18F-FDG PET/MRI performed an paramount role in identification of gynecological cancer lesions but didn’t show superiority when compared to PET/CT [39]. Possible explanation for the unsatisfactory results may be as follows. Firstly, some lesions were microscopic and out of the detection limit [45]. Secondly, inflammation, or concomitant infection led to hypermetabolism of LNs in PET scans, which can be mistakenly recognized to be metastasis from pelvis [46, 47]. Thirdly, MRI couldn't provide precise clear images for movable structures such as lung, which moves up and down with each breath. Thus some lesions that metastasized to movable structures might be omitted by PET/MRI.

The heterogeneity between the included studies was significant in this meta-analysis. Many influencing factors might result in the noted heterogeneity of the included studies. Firstly, different reference standards were taken in different studies. Secondly, the baseline differed among the patients in the included studies. Also, the study qualities were not same. Secondly, some studies didn’t provide sufficient information of the diagnostic performance, the imaging reference standards were not consistent, and the follow up strategy was different

We should acknowledge that this study had several limitations. Firstly, the heterogeneity between the included studies was significant in this meta-analysis. Thirdly, publication bias tests and plots were not performed since the studies included were not enough, and our review was based on the reported results, omitting some possible unpublished studies. Finally, the number of included studies was not large enough.

In conclusion, 18F-FDG PET/MRI, with high sensitivity and high specificity, is a promising imaging method for patient based assessment of pelvic gynecological malignancies, especially for the detection of lymph node metastasis. However, its diagnostic value of lesions in some sites such as pulmonary, is not satisfactory. Histopathological examination is still the gold standard for precise diagnosis. Following these very promising first attempts of PET/MRI for tumor staging of female pelvic malignancies, more studies need to be done.

## Supporting information

S1 Checklist PRISMA Checklist. (DOC)

S1 Fig Risk of bias and applicability concerns graph and summary: review authors' judgements about each domain presented as percentages across included studies. (TIF)

S1 Table Distribution of metastasis and invasion for gynecological malignancies in the studies. (DOCX)
