# Correction: Diagnostic role of 18F-FDG PET/MRI in patients with gynecological malignancies of the pelvis: A systematic review and meta-analysis

**DOI:** 10.1371/journal.pone.0202314

**Published:** 2018-08-09

**Authors:** Ji Nie, Jing Zhang, Jinsheng Gao, Linghong Guo, Hui Zhou, Yuanyuan Hu, Chenjing Zhu, Qingfang Li, Xuelei Ma

The fourth author, Linghong Guo, should be noted as having also contributed equally to this work.
