# Supplementary material for: Diagnostic role of 18F-FDG PET/MRI in patients with gynecological malignancies of the pelvis: A systematic review and meta-analysis
Source: PLoS One. 2017 May 8;12(5):e0175401. doi: 10.1371/journal.pone.0175401 (PMC5421770; doi:10.1371/journal.pone.0175401)
Supplement: S1 Checklist — (DOC) [file pone.0175401.s001.doc]

| **Section/topic** | **#** | **Checklist item** | **Reported on page #** |
| --- | --- | --- | --- |
| **TITLE** | | |  |
| Title | 1 | 1 |  |
| **ABSTRACT** | | |  |
| Structured summary | 2 | 2 |  |
| **INTRODUCTION** | | |  |
| Rationale | 3 | 2 |  |
| Objectives | 4 | 3 |  |
| **METHODS** | | |  |
| Protocol and registration | 5 | 3 |  |
| Eligibility criteria | 6 | 3 |  |
| Information sources | 7 | 3 |  |
| Search | 8 | 3 |  |
| Study selection | 9 | 3 |  |
| Data collection process | 10 | 3 |  |
| Data items | 11 | 4 |  |
| Risk of bias in individual studies | 12 | 4 |  |
| Summary measures | 13 | 4 |  |
| Synthesis of results | 14 | 4 |  |

Page 1 of 2

| **Section/topic** | **#** | **Checklist item** | **Reported on page #** |
| --- | --- | --- | --- |
| Risk of bias across studies | 15 | 4 |  |
| Additional analyses | 16 | 4 |  |
| **RESULTS** | | |  |
| Study selection | 17 | 4 |  |
| Study characteristics | 18 | 4 |  |
| Risk of bias within studies | 19 | 5 |  |
| Results of individual studies | 20 | 5 |  |
| Synthesis of results | 21 | 6 |  |
| Risk of bias across studies | 22 | 5 |  |
| Additional analysis | 23 | 6 |  |
| **DISCUSSION** | | |  |
| Summary of evidence | 24 | 8 |  |
| Limitations | 25 | 9 |  |
| Conclusions | 26 | 9 |  |
| **FUNDING** | | |  |
| Funding | 27 | 9 |  |

*From:*  Moher D, Liberati A, Tetzlaff J, Altman DG, The PRISMA Group (2009). Preferred Reporting Items for Systematic Reviews and Meta-Analyses: The PRISMA Statement. PLoS Med 6(7): e1000097. doi:10.1371/journal.pmed1000097

For more information, visit: **www.prisma-statement.org**.

Page 2 of 2
