# Supplementary figures and images for: Diagnostic role of 18F-FDG PET/MRI in patients with gynecological malignancies of the pelvis: A systematic review and meta-analysis
Source: PLoS One. 2017 May 8;12(5):e0175401. doi: 10.1371/journal.pone.0175401 (PMC5421770; doi:10.1371/journal.pone.0175401)

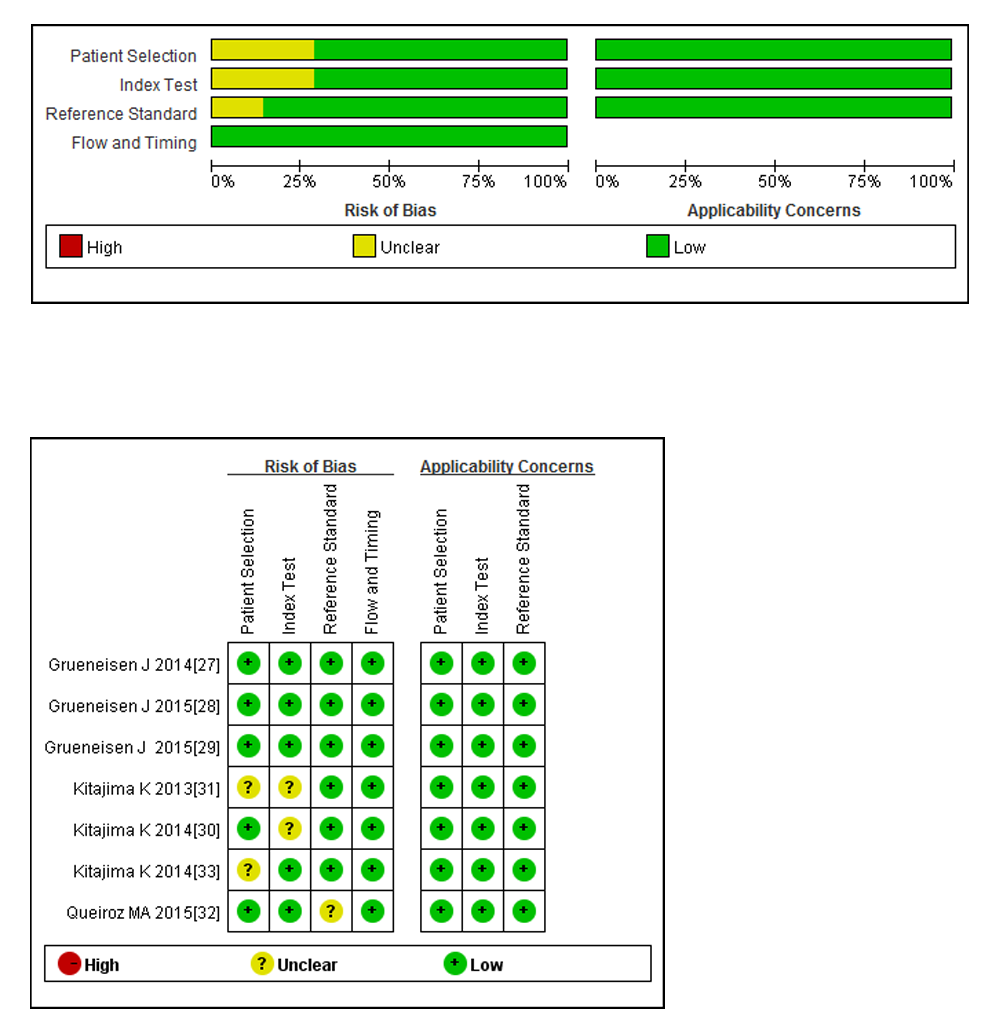

Supplement: S1 Fig — (TIF) [file pone.0175401.s002.tif]
