# Supplementary material for: Diagnostic role of 18F-FDG PET/MRI in patients with gynecological malignancies of the pelvis: A systematic review and meta-analysis
Source: PLoS One. 2017 May 8;12(5):e0175401. doi: 10.1371/journal.pone.0175401 (PMC5421770; doi:10.1371/journal.pone.0175401)
Supplement: S1 Table — (DOCX) [file pone.0175401.s003.docx]

**Diagnostic role of 18F-FDG PET/MRI in Patients with Gynecological Malignancies of the Pelvis: a systematic review and meta-analysis**

Ji Nie, Xuelei Ma, Jing Zhang, Jinsheng Gao , Linghong Guo, Hui Zhou,Yuanyuan Hu,Chenjing Zhu，Qingfang Li

**S1 Table.** Distribution of metastasis and invasion for gynecological malignancies in the studies

|  | Distribution of lesions(malignant/benign) | | | | | | |
| --- | --- | --- | --- | --- | --- | --- | --- |
| Study | Lymph nodes | Peritoneal | Liver | Lung | Bone | Pleural | Primary tumor site |
| Grueneisen J 2014[27] | 40/8 | 15/1 | 7/7 | 3/3 | 3/1 |  | 30/4 |
| Grueneisen J 2015[29] | 35/8 | 23/3 | 5/8 | 8/1 | 5/1 | 2/0 | 3/0 |
